# Efficacy and Safety of Adalimumab as Monotherapy Versus Adalimumab With Methotrexate for Psoriasis: A Systematic Review

**DOI:** 10.7759/cureus.105411

**Published:** 2026-03-17

**Authors:** Ahmed F Alanazi, Muhammad A Almahdi, Shaden O Alqurashi, Saud Alassaf, Abdulaziz Almohanna, Salman T Alayed, Mohammed Alshehri, Kadi S Alsubaie, Salha Hakami

**Affiliations:** 1 College of Medicine, Imam Mohammad Ibn Saud Islamic University, Riyadh, SAU; 2 Faculty of Medicine, Jazan University, Jazan, SAU; 3 College of Medicine, Taif University, Taif, SAU; 4 Department of Dermatology, King Salman Hospital, Riyadh, SAU

**Keywords:** adalimumab, combination therapy, efficacy, humira, methotrexate, psoriasis

## Abstract

Adalimumab is a common biologic used for the management of moderate-to-severe plaque psoriasis. Despite the known importance of adding methotrexate to adalimumab in reducing immunogenicity and improving clinical response, the extent of its benefit remains unclear. This review aimed to systematically compare adalimumab with and without methotrexate, considering their efficacy and safety in the management of psoriasis. This systematic review included randomized controlled trials, long-term follow-up studies, and observational cohorts that investigated adalimumab monotherapy with adalimumab plus methotrexate in adult patients diagnosed with plaque psoriasis. Data on clinical efficacy using Psoriasis Area and Severity Index (PASI), drug survival, and safety outcomes were extracted and summarized. A total of 128 records were initially identified through database searching. After removal of duplicates and screening of titles and abstracts, 45 full-text articles were assessed for eligibility, of which five studies met the predefined inclusion criteria and were included in the final analysis. The studies showed that the addition of methotrexate reduced the antidrug antibody formation and improved the serum level of adalimumab. Additionally, higher early PASI75 achievement was observed at early follow-up periods (approximately 5-16 weeks, depending on study design), with only modest numerical improvements in select studies. Considering safety profiles, the results were comparable between groups, with rare serious adverse events reported. However, the rate of discontinuation because of adverse events was higher with combination therapy. Addition of methotrexate to adalimumab therapy for the management of psoriasis may reduce immunogenicity and optimize the drug pharmacokinetics; however, it does not show a significant advantage considering clinical efficacy or long-term treatment persistence. Therefore, according to the study findings, the combination therapy should be selectively used in patients with suboptimal response or in those with suspected immunogenicity, while retaining the standard use of adalimumab monotherapy for the rest of the patients.

## Introduction and background

Psoriasis is one of the chronic, inflammatory skin disorders that is mainly characterized by the hyperproliferation of keratinocytes and dysregulation of the immune system, causing the formation of scaly and erythematous plaques [1,2]. Psoriasis affects millions of individuals worldwide and has a significant negative impact on their quality of life because of its physical and psychological symptoms [3-5]. Over the years, there has been an expansion of the options available for the management of psoriasis, with biologic therapies such as adalimumab becoming prominent in the management of moderate-to-severe cases of psoriasis [6,7]. Adalimumab is an anti-tumor necrosis factor-alpha (TNF-α) monoclonal antibody that works by inhibiting TNF-α [8,9]. Psoriasis pathogenesis is primarily driven by the interleukin (IL)-23/Th17 immune axis, which promotes keratinocyte proliferation and chronic inflammation. TNF-α also plays a significant role in amplifying the inflammatory cascade and has become an important therapeutic target [10,11].

Methotrexate is a conventional synthetic disease-modifying antirheumatic medication that is often used in combination with biologic treatment to increase its therapeutic outcomes in autoimmune disorders, including psoriasis [12,13]. However, the relative efficacy and safety profile of adding methotrexate to adalimumab in comparison with adalimumab monotherapy remains unclear [14]. Understanding the advantages and potential risks associated with this addition is crucial for optimizing patient care.

Despite the widespread clinical use of adalimumab in psoriasis, uncertainty remains regarding whether the addition of methotrexate provides meaningful improvements in clinical outcomes beyond pharmacokinetic advantages. Existing studies report heterogeneous findings, highlighting the need for a structured synthesis of the available evidence. Hence, the current systematic review aims to evaluate and compare the efficacy and safety profiles of adalimumab as monotherapy with its combination with methotrexate in the management of psoriasis.

## Review

Methodology

This systematic review aimed to assess the efficacy and safety of adalimumab monotherapy compared to adalimumab with methotrexate in the management of psoriasis. The review was conducted following a structured process in accordance with the Preferred Reporting Items for Systematic Reviews and Meta-Analysis (PRISMA) guidelines [15] and was registered on PROSPERO (registration number: CRD420251171542) [16].

The search for relevant studies was conducted across different electronic databases, including CENTRAL, Cochrane Central Register of Controlled Trials, MEDLINE, PubMed, and Scopus, to identify clinical trials and observational studies published until November 2025. The search was conducted using different MeSH terms and keywords, including “adalimumab,” “Methotrexate,” “psoriasis,” “monotherapy,” and “Combination therapy,” which were used in different combinations to capture a wide range of included studies.

Inclusion criteria included randomized controlled studies, observation studies, studies conducted among adult patients diagnosed with moderate-to-severe psoriasis, studies conducted on comparison of the efficacy and safety of adalimumab monotherapy with adalimumab combined with methotrexate, studies published in English, studies with full-text access, and studies providing at least one of the following outcomes: Psoriasis Area and Severity Index (PASI) scores, which is a widely used scoring system ranging from 0 to 72 that evaluates psoriasis severity based on lesion erythema, thickness, scaling, and body surface area involvement; quality of life; or adverse events. Exclusion criteria included non-randomized controlled studies, studies involving other biologic therapies, and studies that did not report relevant outcomes.

Data extraction was conducted independently by two reviewers to ensure accuracy and minimize possible bias. The extracted data included study design, patient characteristics, treatment regimens, duration of treatment, and efficacy outcomes, as well as safety profile. Efficacy outcomes included results of PASI scores and improvement in the overall severity of the condition, while the safety profile included the incidence of adverse events associated with the treatment. Any discrepancies between the two reviewers in the extraction of the data were resolved through discussion or via consultation with a third reviewer.

The quality of the included studies was assessed using different tools according to the design of the studies, mainly the Cochrane Risk of Bias [17] tool for randomized controlled trials and the Newcastle-Ottawa Scale [18] for observational studies.

Results

**Figure 1:**
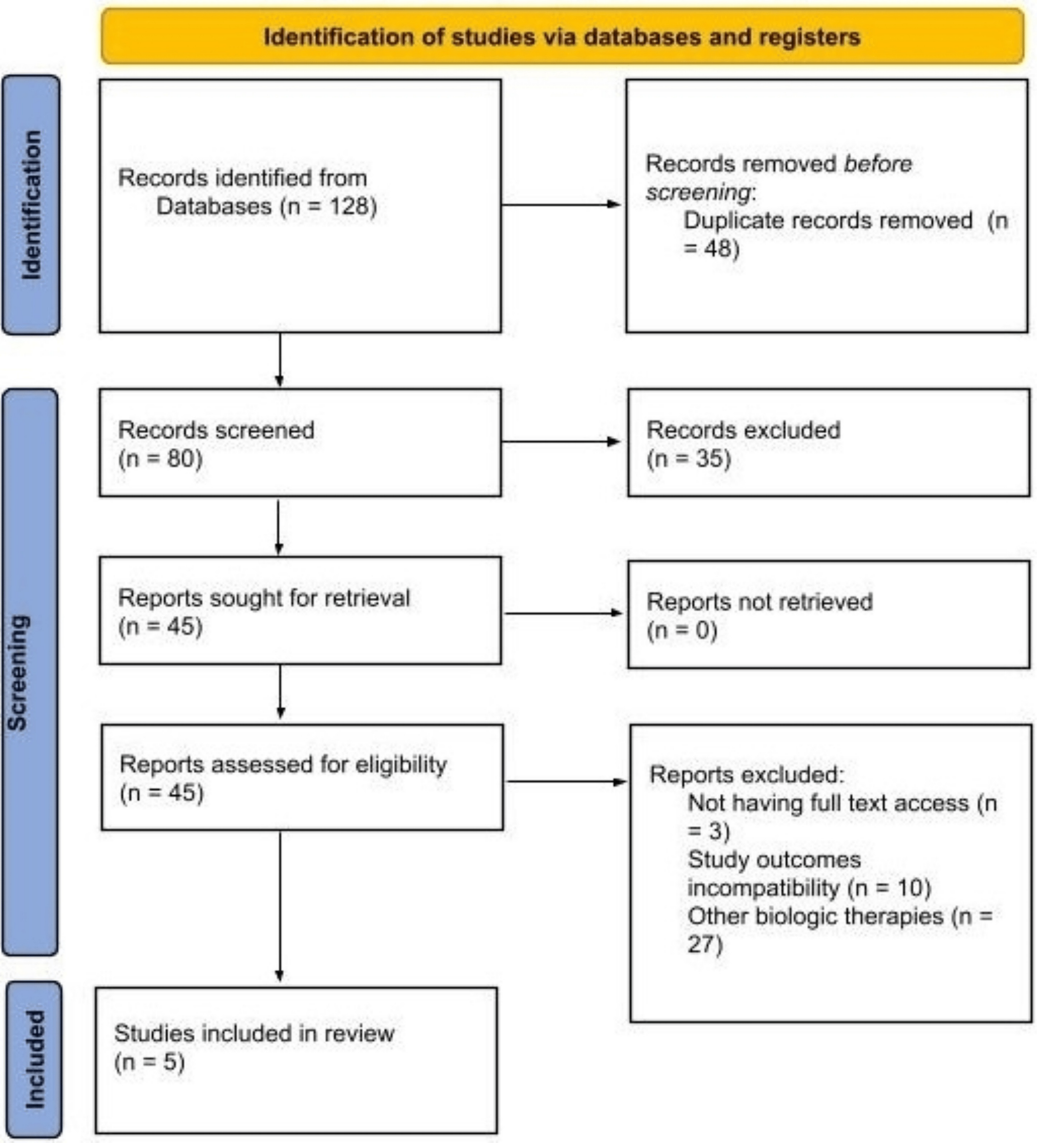
Preferred Reporting Items for Systematic Reviews and Meta-Analysis (PRISMA) flowchart.

The review included five studies (Figure 1) [19-23] with a heterogeneous body of evidence. The study designs ranged from randomized controlled trials [19,21], registry-based comparative cohorts [20], and prospective observational studies [22], with one phase IIIb open-label trial [23]. Most of the included studies were conducted in European settings [19-22], including the Netherlands [19-22] and the United Kingdom [20], with one study conducted in Canada [23]. Sample size of the studies varied from fewer than 50 patients [22,23] to over 1,700 patients [21]. The follow-up durations ranged between 12 and 24 weeks in short-term assessment and up to three years in long-term observation studies (Table 1).

**Table 1:** Characteristics of the included studies comparing adalimumab monotherapy versus adalimumab with methotrexate in psoriasis. ADL: adalimumab; MTX: methotrexate; EOW: every other week; RCT: randomized controlled trial

Study (year)	Study design	Setting/Country	Population	Sample size	Follow-up duration	Intervention comparison
Kraaij et al. (2022) [19]	Randomized controlled trial	The Netherlands	Adults with chronic plaque psoriasis	61 randomized (31 ADL + MTX, 30 ADL)	49 weeks	ADL + MTX 10 mg/week vs. ADL monotherapy
Yiu et al. (2025) [20]	Target trial emulation (registry-based cohort)	UK	Adults with plaque psoriasis	1,784 (231 ADL + MTX, 1,553 ADL)	Up to 3 years	ADL EOW + clinician-determined MTX vs. ADL monotherapy
Huizen et al. (2023) [21]	Multicenter RCT with long-term follow-up	The Netherlands and Belgium	ADL-naïve moderate–severe plaque psoriasis	61 initial; 37 long-term	Up to 145 weeks	Initial ADL + MTX vs. ADL monotherapy
Reek et al. (2012) [22]	Prospective observational cohort	The Netherlands	Psoriasis with inadequate ADL response	45 patients (64 treatment episodes)	Up to 24 weeks	MTX addition and/or ADL dose escalation
Papp et al. (2022) [23]	Phase IIIb open-label single-arm study	Canada	Suboptimal responders to ADL	46	24 weeks	Addition of MTX to ongoing ADL

Among the included studies, the clinical efficacy outcomes showed generally comparable long-term efficacy between the two groups, with some evidence of enhanced early response and higher response rates in the combination therapy. Short-term outcomes showed that methotrexate addition could accelerate the treatment response, as higher early PASI75 achievement was observed at early follow-up periods (approximately 5-16 weeks, depending on the study design) [19,23]. However, at longer follow-up (up to 145 weeks), PASI75 response rates were typically between one-third and one-half of the patients who received adalimumab monotherapy [19-21]. Long-term registry data indicated that PASI75 response rates declined over time in both groups, with overlapping confidence intervals between monotherapy and combination therapy at one and three years [20]. Drug survival followed a similar pattern, with modestly higher persistence observed in the combination therapy groups across studies, but without consistent statistically significant differences [19-21]. In populations with inadequate initial response to adalimumab, methotrexate add-on therapy was associated with modest improvements in PASI scores, although response rates remained limited and clinically heterogeneous [22]. Single-arm evidence further supported clinically meaningful within-group PASI improvements following methotrexate addition, particularly for partial responders [23] (Table 2).

**Table 2:** Summary of efficacy outcomes. ADL: adalimumab; MTX: methotrexate; PASI: Psoriasis Area and Severity Index

Study	Follow-up	ADL monotherapy	ADL + MTX	Between-group comparison
Kraaij et al. (2022) [19]	Week 49	PASI75: 36.7%; PASI90: 16.7%; drug survival: 58.6%	PASI75: 58.1%; PASI90: 29.0%; drug survival: 74.2%	PASI75 (p = 0.13); PASI90 (p = 0.34); drug survival (p = 0.15)
	Week 5	PASI75: 3.5%	PASI75: 22.6%	P = 0.05
Yiu et al. (2025) [20]	1 year	PASI75: 52.0%; drug survival: 78.1%	PASI75: 49.4%; drug survival: 79.1%	PASI75 Δ −2.5% (95% CI = −21.0 to 15.9); survival Δ +1.0%
	3 years	PASI75: 32.4%	PASI75: 37.2%	Δ +4.9% (95% CI = −16.1 to 25.7)
Huizen et al. (2023) [21]	Week 109	PASI75: 50.0% (8/16); drug survival: 41.4%	PASI75: 82.3% (14/17); drug survival: 54.8%	PASI75 favoring ADL + MTX; drug survival (p = 0.326)
	Week 145	PASI75: 60.0% (9/15); drug survival: 41.4%	PASI75: 64.3% (9/14); drug survival: 51.6%	Drug survival (p = 0.464)
Reek et al. (2012) [22]	Week 12	PASI50: 25% PASI75: 0% PASI mean: 1.6	PASI50: 9% PASI75: 9% PASI mean: 1.2	Descriptive only
	Week 24	PASI50: 35% PASI75: 9% PASI mean: 2.5	PASI50: 18%; PASI75: 9% PASI mean: 1.1	No significant improvement
Papp et al. (2022) [23]	Week 16	Baseline PASI: 10.4	PASI50: 43–65%; PASI75: 28–39%	Within-group improvement (p < 0.001)
	Week 24		PASI90: 13–28%; PASI100: 13–26%	Single-arm study

With no consistent rise in severe side effects related to methotrexate co-administration, safety outcomes were essentially similar between adalimumab monotherapy and combination therapy with methotrexate [19-23]. Overall, rates of negative events were comparable across treatment modalities in trials; serious adverse events were uncommon and usually unrelated to study drugs [19-23]. However, some studies reported a higher rate of treatment discontinuation due to adverse events. Commonly reported adverse events included mild infections, injection-site reactions, headache, gastrointestinal discomfort, and transient liver enzyme elevations associated with methotrexate therapy [21-30] in the combination therapy groups, suggesting potential tolerability considerations with prolonged methotrexate use [21]. In contrast to safety findings, pharmacokinetic and immunogenicity outcomes consistently favored combination therapy [19-21]. Improved pharmacologic stability was shown by patients on methotrexate co-therapy, as evidenced by a lower proportion of patients with subtherapeutic medication trough levels and less formation of anti-adalimumab antibodies [19,20]. Higher or more stable serum adalimumab concentrations were seen in patients receiving methotrexate co-therapy, and less immunogenicity was often linked to better or sustained clinical response [19,23] (Table 3). Clinical response outcomes such as PASI scores were evaluated at predefined follow-up intervals across studies, typically ranging from 5 weeks to 145 weeks, depending on study design and follow-up duration [19-21].

**Table 3:** Safety, pharmacokinetics, and immunogenicity outcomes. ADL: adalimumab; MTX: methotrexate; AE: adverse event; SAE: serious adverse event; ADA: antidrug antibody

Study	Safety outcomes	Pharmacokinetics and immunogenicity
Kraaij et al. (2022) [19]	No SAEs; similar AE rates between groups	Significantly fewer ADAs with ADL + MTX; higher serum trough levels
Yiu et al. (2025) [20]	No difference in SAEs	Lower ADA levels in the ADL + MTX group; similar ADL concentrations
Huizen et al. (2023) [21]	More discontinuations due to AEs in the combination group; SAEs rare and unrelated	Fewer ADA and fewer subtherapeutic trough levels with MTX co-therapy
Reek et al. (2012) [22]	No MTX-related SAEs	Pharmacokinetics not formally assessed
Papp et al. (2022) [23]	No new safety signals; one SAE deemed unrelated	Higher ADA trough concentrations associated with better clinical response

Discussion

The current systematic review investigated the available evidence comparing adalimumab monotherapy with the combination of adalimumab with methotrexate in the management of plaque psoriasis. The review showed that while concomitant use of methotrexate with adalimumab can be associated with modulated pharmacologic parameters and immunogenicity, it does not confer a clear clinical advantage over monotherapy of adalimumab in the broader psoriasis population. In terms of clinical efficacy, combination therapy was associated with numerically higher response rates and was associated with greater improvement of disease control in some studies, especially at earlier time points [19,23]. These results are similar to the rationale that suppression of antidrug antibody formation may be associated with preserving biologic activity and improving the early therapeutic response [24,25]. Reduced antidrug antibody formation and more favorable pharmacokinetics have been reported in the combination therapy of methotrexate plus adalimumab, which theoretically could improve exposure to the medications and clinical outcomes. Immunosuppressive properties of methotrexate are thought to attenuate the humoral response of immunity against biologic agents; therefore, they could mitigate the immunogenicity and prolong the drug survival [26,27]. This was reported in previous studies among patients with other inflammatory diseases, where antidrug antibody formation correlated with reduced biologic efficacy and shorter treatment persistence [28-30]. However, the current review showed that the magnitude of these mechanistic benefits in psoriasis has not translated into significant superiority in the clinical outcomes, including PASI75 and long-term drug survival in comparison with adalimumab monotherapy.

In addition, the longitudinal outcomes indicated that standard clinical efficacy measures, such as PASI75, are similar between the two groups at one year and beyond. In a previous study, the results showed that adalimumab in combination with methotrexate did not reveal significant differences in drug survival or PASI75 responses compared with monotherapy [20]. However, another systematic review conducted by Xie et al. showed that a combination of methotrexate with biologic therapy is associated with better efficacy in the treatment of psoriasis with no increased risk of severe adverse events or drug withdrawals because of adverse events [31]. In addition, another study conducted among patients with rheumatoid arthritis showed contrasting results, where combination therapy was shown to yield more clinical benefits with TNF inhibitors [30,31].

In addition, the current review showed that the safety profile of adalimumab with and without methotrexate was generally comparable across the included studies [19-23]. Serious adverse events were rare in both groups and not clearly associated with either treatment strategy; however, there is a slightly higher incidence of discontinuations because of tolerability concerns in patients treated with combination therapy [21]. These results are similar to what was reported in previous studies showing that methotrexate can be associated with cumulative adverse events, including hepatic enzyme elevations and cytopenias, especially with prolonged use [32].

Limitations

A notable limitation is the small sample sizes in several of the included studies. For instance, multiple analyzed trials and prospective cohorts involved fewer than 50-65 patients [19-23]. Such limited sample sizes reduce the statistical power required to detect subtle but clinically meaningful differences in efficacy outcomes, such as PASI75 response rates and long-term drug survival, between adalimumab monotherapy and combination therapy. Additionally, small patient cohorts restrict the ability to accurately evaluate the incidence of rare but severe adverse events, potentially limiting the robustness of the comparative safety profiles. When coupled with the overall heterogeneity of the study designs and populations, these sample size constraints restrict the broad generalizability of the findings.

## Conclusions

The current review showed that routine addition of methotrexate to the treatment protocol of adalimumab for all patients with psoriasis may not be associated with substantial clinical advantages beyond the immunogenicity profile. Furthermore, it is important to emphasize the safety trade-off; the increased risk of adverse event-related discontinuations reinforces adalimumab monotherapy as a safer and more sustainable standard of care for the broader patient population. Instead, the combination therapy should be used selectively among patients where immunogenicity is suspected to undermine the response, or in patients with a suboptimal initial response.
